# Euphejolkinolide A, a new *ent*-abietane lactone from *Euphorbia peplus* L. with promising biological activity in activating the autophagy-lysosomal pathway

**DOI:** 10.1016/j.heliyon.2023.e13691

**Published:** 2023-02-13

**Authors:** Xiaoqian Ran, Qing-Yun Lu, Ying-Yao Li, Xue-Xue Pu, Yarong Guo, Ming-Rui Yuan, Shi-Peng Guan, Mao Sun, Lijin Jiao, Yong-Gang Yao, Ying-Tong Di, Xiao-Jiang Hao, Rongcan Luo

**Affiliations:** a Key Laboratory of Animal Models and Human Disease Mechanisms of the Chinese Academy of Sciences & Yunnan Province, KIZ-CUHK Joint Laboratory of Bioresources and Molecular Research in Common Diseases, Kunming Institute of Zoology, Chinese Academy of Sciences, Kunming, 650204, China; b State Key Laboratory of Phytochemistry and Plant Resources in West China, Kunming Institute of Botany, Chinese Academy of Sciences, Kunming, 650201, China; c Kunming College of Life Science, University of Chinese Academy of Sciences, Kunming, Yunnan, 650204, China; d College of Life Sciences, Yunnan University, Kunming, 650091, China; e College of Traditional Medicine, Yunnan University of Chinese Medicine, Kunming, 650500, China; f School of Life Sciences, Division of Life Sciences and Medicine, University of Science and Technology of China, Hefei, 230026, China; g CAS Center for Excellence in Brain Science and Intelligence Technology, Chinese Academy of Sciences, Shanghai, 200031, China; h Yunnan Key Laboratory of Natural Medicinal Chemistry, Kunming, Yunnan, 650201, China; i Guizhou Chemical Drug Research and Development Engineering Technical Center, Guizhou Medicinal University, Guiyang, 550004, China

**Keywords:** *Euphorbia peplus* L., *Euphorbiaceae*, *Ent*-abietane diterpenoid, Euphejolkinolide A, Autophagy-lysosomal pathway

## Abstract

A new *ent*-abietane diterpenoid, named Euphejolkinolide A (**1**), was isolated from the whole plant of *Euphorbia peplus* L. Its structure, including absolute configurations, was determined by spectroscopic analyses and was corroborated by single-crystal X-ray diffraction analysis. This new compound was assessed for its activity to induce lysosome biogenesis through Lyso-Tracker Red staining, in which compound **1** could significantly induce lysosome biogenesis. In addition, quantitative real-time PCR (qRT-PCR) analysis demonstrated a direct correlation between the observed lysosome biogenesis and the transcriptional activation of the lysosomal genes after treatment with the compound **1**. Moreover, compound **1** promoted autophagic flux by upregulating LC3-II and downregulating SQSTM1 in both human microglia cells and U251 cells, which is required for cellular homeostasis. Further results suggested **1** induced lysosome biogenesis and autophagy which was mediated by TFEB (transcription factor EB). The structure activity relationships (SAR) analysis suggested that the carbony1 at C-7 in **1** might be a key active group. Overall, the current data suggested that **1** could be a potential compound for lysosome disorder therapy by induction of autophagy.

## Highlights

• One undescribed *ent*-Abietane lactone was obtained from *Euphorbia peplus* L.
• The mCherry-GFP-LC3 protein was combined with flow cytometry analysis to directly reflect the strength of autophagic flux.
• Compound **1** activates the autophagy-lysosome system mediated by TFEB (transcription factor EB).
• Carbonyl at C-7 in compound **1** as a nucleophilic group might be a key active group.

## Graphical abstract

**Figure:**
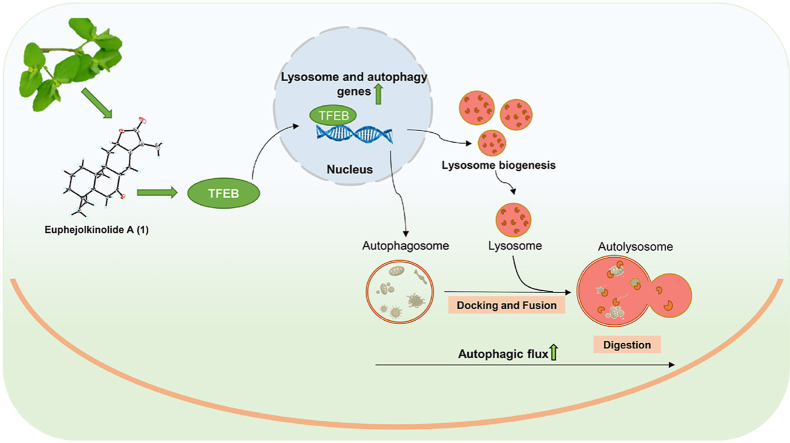

## 1 Introduction

Macroautophagy, herein referred to as autophagy (which means ‘self-eating’) refers to the catabolic process in which cellular components are degraded by the machinery of the lysosome [1]. Autophagy dysfunction has been implicated in the pathogenesis of many neurodegenerative diseases [1,2]. It was reported that autophagy has an essential role against the development of a number of neurodegenerative diseases [[3], [4], [5], [6]]. Previously, we found that peroxisomal ACAA1 p. N299S mutation contributes to Alzheimer's disease by disturbing autophagy-lysosomal pathway (ALP) [7]. In addition, we found gemfibrozil, a U.S. Food and Drug Administration (FDA)-approved drug primarily used to treat hyperlipidemia [8] that could reduce amyloid-β (Aβ) pathology by activating ALP [9]. Therefore, induction of ALP may serve as a viable therapeutic target for the treatment of neurodegenerative diseases [2,[10], [11], [12], [13]].

*Euphorbia peplus* L. (Euphorbiaceae, *E. peplus*) is a small, annual, herbaceous plant with milky latex [14]. This plant is known to produce a large variety of diterpenoids, some of which are highly irritant and have tumor-promoting activity, while others exhibit antileukemic, cytotoxic, and analgetic activity [15,16]. *E. peplus* is rich of diterpenoids, triterpenoids, coumarin and acetophenone. These diterpenoids can be divided into different types according to their core frameworks, such as abietanes, lathyranes, kauranes, mytstinols, atisanes, ingenes, neo-clerodanes, and clerodanes et, al [15,17]. Due to broad-spectrum biological activity and rich structural diversity (multiple types of ring systems, high degree of oxidation, complex types of substituents, etc.), *Euphorbia* diterpenoids have long been the focus of natural medicine research [15,[17], [18], [19], [20], [21], [22], [23], [24]]. For example, we found that 20-deoxygenol 5-angelate from *E. peplus* and its analogous could induce lysosome biogenesis and clear Aβ in the brain of mice, suggesting that these compounds have the potential to treat neurodegenerative diseases [25]. In addition, we also examined the effects of cyclojatrophanes A-C and euphopepluanones A-E, another diterpenoids isolated from *E. peplus* on lysosomal-autophagy pathway [20,26,27]. *Ent*-Abietanes with a lactone ring are considered as the other main bioactive compounds in many species of the genus of *Euphorbia*. For instance, jolkinolide B and its derivatives are well-known for their anti-tumor activity [19,[28], [29], [30], [31], [32]].

In this study, one undescribed *ent*-abietane, named Euphejolkinolide A (**1**) was obtained from the whole plant of *E. peplus*, which could induce lysosome biogenesis and activate ALP. Our findings suggest that **1** has promising biological activity for neurodegenerative diseases therapy by induction of autophagy.

## 2 Materials and methods

### 2.1 General experimental design

Nuclear magnetic resonance (NMR) spectra (Figs. S1 and S2) were measured on AVANCE III 500 MHz NMR spectrometers (Bruker BioSpin Group, Faellanden, Switzerland) with the solvent signals (CDCl_3_: *δ*_H_ 7.26/*δ*_C_ 77.0) as the internal standard. Circular dichroism (CD) spectra (Fig. S8) were determined on the Applied Photophysics circular dichroism spectrometer (Applied Photophysics, Leatherhead, Surrey, UK). Optical rotation measurements were conducted with a Jasco P-1020 automatic polarimeter. Infrared radiation (IR) spectra (Fig. S10) were recorded on a NICOLET iS107 Mid-infrared spectrometer. X-ray single crystal diffraction experiment was performed on a SuperNova, Dual, Cu at 0, AtlasS2 diffractometer. High-resolution Electron Spray Ionization Mass spectrometry (HRESIMS) data were recorded on an Agilent 1290 UPLC/6540 Q-TOF mass spectrometer in positive mode. An Agilent 1260 series instrument equipped with a SunFire-C18 column (5 μm, 10 * 250 mm) was used for high-performance liquid chromatography (HPLC) Semi-preparation (λ = 280 nm; CH_3_CN/H_2_O with isocratic elution, 60:40; 20 min). Silica gel (100–200, 200–300, 300–400) mesh (Qingdao Marine Chemical, Inc.), and Sephadex LH-20 (20 * 1500 mm, Pharmacia) were used for column chromatography (CC).

### 2.2 Plant material

The whole plant organs of *E. peplus* were collected in July 2018 from Kunming Botanical Garden, Kunming Institute of Botany, Yunnan Province, China (location: 102° 44′E, 25° 07′ N, at an altitude of 1900 m). The plant was identified by Prof. Hu Shi-Jun (Kunming Institute of Botany, Chinese Academy of Sciences) [33]. A voucher specimen (no. kep-09-18) has been deposited in the herbarium of the Kunming Institute of Botany, Chinese Academy of Science.

### 2.3 Extraction and isolation

The air-dried whole plant organs of *E. peplus* (60 kg) were extracted with methanol (room temperature) thrice. The crude extract (3 kg) was obtained by reflux, and was subjected to a silica gel (10 kg) column and eluted with petroleum ether/ethyl acetate (100:0, 50:1, 20:1, 10:1, 3:1, and 0:100, *v/v*, 10 L/each gradient) gradient to obtain 4 fractions, F1 – F4. Fraction F2 (500 g) was further subjected to MCI gel (2.0 kg) treatment with MeOH–H_2_O (40:60, 60:40; 80:20, and 100:0, *v/v*, 12 L/each gradient) to obtain 15 subfractions, F2-1 - F2-15. Traction F2-5 (2 g) was purified by silica gel column (80 g) chromatography, using petroleum ether/ethyl acetate (20:1, 10:1, 5:1, 3:1, and 1:1, *v/v*, 1 L/each gradient) to obtain F2-5-11 (500 mg), and F2-5-11 (500 mg) was further treated with Sephadex LH-20 (MeOH/CH_2_Cl_2_, 50:50, *v/v*) to obtain 6 subfractions, F2-5-11-1 - F2-5-11-6. Then F2-5-11-5 (45 mg) was passed through preparative HPLC (CH_3_CN/H_2_O, 60:40, *v/v*) to afford Euphejolkinolide A (**1**, 10 mg).

**Euphejolkinolide A (1)** Monoclinic crystal; C_20_H_28_O_3_; [α]_D_^25^ +11 (*c* 0.07, MeOH); UV (MeOH) *λ*_max_ (log *ε*) 230 (3.44) nm; ECD (MeOH) *λ*_max_ (Δε) 232 (7.92) nm; IR (KBr) ν_max_ 3300−2500, 3466, 2930, 2872, 1741, 1619, 1452, 1371, 1276, 1238, 1114, 1027, and 714 cm^−1^; ^1^H NMR data (600 MHz, CDCl_3_), see Table 1; positive-ion mode HRESIMS *m/z* 317.2112 [M + H]^+^ (calcd for C_20_H_29_O_3_, 317.2111).

**Table 1:** ^1^H and^13^C NMR Data of 1 in CDCl_3_ (*δ* in ppm and *J* in Hz) a.

No.	*δ*_H_	*δ*_C_	No.	*δ*_H_	*δ*_C_
1a	1.84 m	38.8 t	10	─	37.2 s
1b	1.00 dd (13.1, 4.1)		11a	2.58 ddd (11.9, 5.9, 2.7)	33.4 t
2a	1.63 m	18.5 t	11b	1.11 dd (11.9)	
2b	1.57 m		12	4.54 dd (11.9, 5.9)	79.3 d
3a	1.51 m	41.5 t	13	─	160.8 s
3b	1.20 td (13.1, 4.1)		14a	3.09 dd (12.7, 2.0)	25.5 t
4	─	33.7 s	14b	2.23 (o)	
5	1.32 dd (14.4, 2.9)	54.5 d	15	─	120.7 s
6a	2.50 dd (14.4, 2.9)	39.7 t	16	─	174.4 s
6b	2.34 t (14.4)		17	1.81 s	8.3 q
7	─	209.6 s	18	0.89 s	21.2 q
8	2.23 (o)	49.5 d	19	0.87 s	32.9 q
9	1.39 td (11.9, 2.7)	52.4 d	20	1.04 s	13.6 q

a Recorded at 500 MHz (^1^H) and 125 MHz (^13^C); o: overlapped.

### 2.4 X-ray single-crystal structure analysis of compound **1**

Crystal data (Table S1) for compound **1**: C_20_H_28_O_3_, *M* = 316.42, *a* = 7.1902(2) Å, *b* = 9.1117(3) Å, *c* = 13.5961(3) Å, *α* = 90°, *β* = 105.2350(10)°, *γ* = 90°, *V* = 859.44(4) Å3, *T* = 100.(2) K, space group P_212121_, *Z* = 2, *μ*(Cu Kα) = 0.635 mm^−1^, 15218 reflections measured, 15218 independent reflections (R_int_ = 0.0424). The final R_*1*_ values were 0.0304 (*I* > 2*σ*(*I*)). The final w*R*(F^2^) values were 0.0802 (*I* > 2*σ*(*I*)). The final R_*1*_ values were 0.0312 (all data). The final w*R*(F^2^) values were 0.0813 (all data). Flack parameter = 0.12(8).

### 2.5 RNA extraction and quantitative real-time PCR (qRT-PCR)

Total RNA was extracted from U251 cells using RNA simple Total RNA Kit (DP419; TIANGEN, Beijing, China) and the quality of total RNA was measured on a biophotometer (Eppendorf). About 1 μg total RNA which meet the requirements with an A260/A280 ratio of 1.8–2.0 was used to synthesize cDNA by using oligodT_18_ primer and Moloney murine leukemia virus (M-MLV) reverse transcriptase (M1701; Promega). qRT-PCR was conducted using iTaq Universal SYBR Green Supermix (172–5125; Bio-Rad Laboratories) with gene-specific primer pairs (Table S2) on a CFX Connect Real-Time PCR Detection System (Bio-Rad Laboratories, Hercules, CA). The thermal cycling protocol was one cycle at 95 °C for 5 min, 40 cycles of 95 °C for 20 s, 58 °C for 20s and 72 °C for 20 s, one cycle at 95 °C for 1 min and 58 °C for 1 min. The GAPDH transcript was used for the normalization of the target gene.

### 2.6 Lyso-Tracker Red staining

The ability of compound **1** in activating lysosome biogenesis was evaluated through Lyso-Tracker Red staining as previously described [25]. Briefly, HM cells and U251 cells were described in our previous study [7,9,34,35] and were cultured in Dulbecco's Modified Eagle Medium (DMEM) and Roswell Park Memorial Institute 1640 medium, respectively, supplemented with 10% fetal bovine serum (Gibco-BRL, 10099–141) at 37 °C incubator with 5% CO_2_ and 95% humidity. The cells cultured in Lab-Tek II Chamber Slide (Thermo Fisher Scientific, 154526) were treated with compound **1** for 24 h and then stained by Lyso-Tracker red (500 nM) for 1 h [25]. For better live-cell imaging, glass bottom dishes (NEST, 801001) were also used in cell growing through an Olympus FluoView™ 1000 confocal microscope (Olympus, America). Images were analyzed with FV10-ASW 2.1 Viewer.

### 2.7 Western blot

The HM and U251 cells were cultured in 6-well plates. Western blotting for target proteins was performed using the common approach as described in our previous studies [9,36]. In brief, a protein lysis buffer (Beyotime Institute of Biotechnology, P0013) was used to prepare cell lysates. The protein concentration was determined using the BCA protein assay kit (Beyotime Institute of Biotechnology, P0012). About 20 μg total proteins were separated by 12% sodium dodecyl sulfate polyacrylamide gel electrophoresis and were transferred to polyvinylidene difluoride membrane (Bio-Rad, L1620177 Rev D). The membrane was blocked in 5% (*w:v*) skim milk at room temperature for 2 h. The membrane was incubated with primary antibody against CTSB (1:1000; Affinity, AF5189), SQSTM1 (1:1000, Elabscience, E-AB-62289), LC3 (1:1000, Proteintech, 14600-1-AP), β-Tubulin (1:50000, Affinity, DF7967), at 4 °C overnight respectively. The membrane was then washed 3 times with TBST (Tris buffered saline [Cell Signaling Technology, 9997] with 0.1% Tween 20 [Sigma, P1379]) for 5 min each time, and then incubated with either peroxidase-conjugated anti-rabbit IgG (KPL; 5220–0458; 1:10000) at room temperature for 1 h. The epitope was visualized using ECL Western Blot Detection Kit (Millipore, WBKLS0500). Western blot of β-tubulin was used as an inner control for measuring the protein level of the target gene. The densitometry of target proteins was evaluated by ImageJ software (National Institutes of Health, Bethesda, Maryland, USA).

### 2.8 Flow cytometry analysis

HM mCherry-GFP-LC3 cells with stably expressing of a triple fusion protein (red fluorescent protein (mCherry), green fluorescent protein (GFP) and the autophagosome marker LC3 [36]), which can directly reflect the strength of autophagic flux, were used in detecting the bioactivities of compound **1** and other compounds. These cells show yellow fluorescence due to the co-expression of red mCherry and green GFP in the absence of autophagy. When autophagy process goes well, autophagosomes and lysosomes fuse to form autolysosomes, and the acidic lysosomal environment quenches the fluorescence of acid-sensitive GFP, while mCherry is not affected, and then the autolysosomes show red fluorescence. Therefore, red fluorescence in the cells can indicate the formation of autolysosome [37,38]. The higher red fluorescence and the lesser green fluorescence, the better the flux smooth from autophagosome to autolysosomes. HM mCherry-GFP-LC3 cells were cultured in DMEM supplemented with 10% fetal bovine serum (Gibco-BRL, 10099–141) at 37 °C incubator with 5% CO_2_ and 95% humidity. HM mCherry-GFP-LC3 cells were cultured in 12-well plates for 24 h, and the compound was added directly to the culture medium (10 μM and 40 μM). After 24-h treatment, the cells were harvested and fixed by 4% PFA (paraformaldehyde). The fixed cells then were followed by a flow cytometry test to check the autophagic flux. Data were analyzed using FlowJo software (FLOWJO, LLC).

### 2.9 Confocal laser scanning assay

The HM mCherry-GFP-LC3 cells were cultured in glass-bottom cell dish (NEST, 801001) overnight. The cells were fixed by 4% PFA after the 24 h treatment of compound **1** or compound **2**–**8**, and then the individual cells were pictured under the Olympus FluoView™ 1000 confocal microscope (Olympus, America). Exception of a shorter treatment with compound **1** for 6 h, the HM TFEB-GFP cells were handled the same way. Images were analyzed with FV10-ASW 2.1 Viewer.

### 2.10 Construction of the HM cells with stably expressing of TFEB gene

The HM cells were introduced from the Kunming Cell Bank, Kunming Institute of Zoology, Chinese Academy of Sciences and were maintained in DMEM supplemented with 10% FBS (Gibco, USA, 10099–141) at 37 °C incubator with 5% CO2 and 95% humidity. The coding region of the *TFEB* gene with flag tag was cloned into pLVX-Puro Vector. The response lentivirus system was composed of pLVX-TFEB constructs, packaging plasmid psPAX2 (Addgene, England, 12260) and envelope plasmid PMD2. G (Addgene, England, 12259). The lentivirus supernatant was produced from the HEK293T cells and was used to infect HM cells with the ratio of 4:1 for the response lentivirus and the regulator lentivirus. Infected HM cells were selected in growth medium with 1 μg/mL puromycin.

### 2.11 Statistics and reproducibility

Data analyses were carried out by using GraphPad Prism 8 (GraphPad Software, Inc., La Jolla, CA, USA). The one-way ANOVA (analysis of variance) was performed using the Dunnett's *post hoc* test for comparison between the treated group and control group, and the values were expressed as mean ± standard deviation (SD). It is considered to be statistically significant if a *P* value < 0.05. *, *P* < 0.05; **, *P* < 0.01; ***, *P* < 0.001; ****, *P* < 0.0001.

## 3 Results and discussion

### 3.1 Structural elucidation of compound **1**

**Fig. 1:**
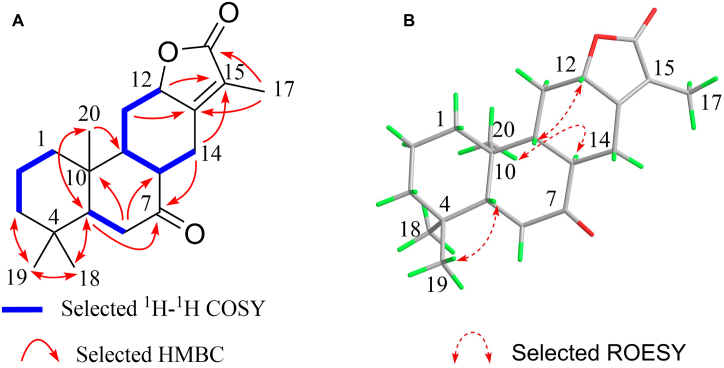
Key HMBC, ^1^H–^1^H COSY, and ROESY correlations structure of **1**. A. Selected ^1^H–^1^H COSY correlations and HMBC correlations of **1**. B. The key ROESY correlations of **1**.

Compound **1** (**Euphejolkinolide A**) was obtained as a colorless monoclinic crystal. Its molecular formula was established to be C_20_H_28_O_3_ by positive HR-ESI-MS (*m/z* 317.2112 [M + H] ^+^, calcd. 317.2112), with 7° of unsaturation (DOU). The ^13^C NMR (Table 1) and DEPT (Figs. S1–3) spectra of compound **1** revealed the presence of 20 carbon resonances, including four tertiary methyls (*δ*_H_ 0.87, 0.89, 1.04, and 1.81, *δ*_C_ 8.3, 13.6, 21.2, and 32.9), six methylene groups, four methine groups (one oxygenated), and six quaternary carbons (one ester carbonyl carbon, one carbonyl carbon, and two tetrasubstituted oleﬁnic carbons), corresponding to three DOU and requiring a tetracyclic structure for compound **1** to complete the DOU count. The IR band at 1741 cm^−1^ and the detection of **1** the downfield H resonance of an oxymethine at *δ*_H_ 4.54 (H-12) and of the ^13^C NMR resonance of a carbonyl at *δ*_C_ 174.4 (s, C-16) suggested the presence of an α,β-unsaturated δ-lactone. This, and the pattern of the methyl multiplicities suggested that compound **1** belongs to *ent*-abietane diterpenoid. Complete assignments of proton and carbon signals of **1** were performed by 2D NMR experiments. As shown in Fig. 1A, correlation analysis on the ^1^H–^1^H correlation spectroscopy (COSY) and heteronuclear singular quantum correlation (HSQC) spectra (Fig. S4) of **1** indicated the presence of three fragments (C-1/C-2/C-3, C-5/C-6, and C-12/C11/C9/C8/C-14). The ^1^H detected heteronuclear multiple bond correlation (HMBC) correlations readily established A and B rings, which were the same as those of 8*α*,14-dihydro-7-oxo-jolkinolide E [39]. In the HMBC spectrum (Fig. S5), cross-peaks of Me-17/C-13, C-15, and C-16, and H-12/C-15 showed the presence of the *α, β*-unsaturated *γ*-lactone ring D, including C-12, 13, 15 and 16. Moreover, HMBC correlations of H_2_-14/C-12 and C-15, and H_2_-11/C-13 and the fragment from C-12 to C-14 established ring C as shown. The planar structure of **1** was thus elucidated as indicated.

The relative configuration of **1** was fixed by nuclear Overhauser effect spectroscopy (ROESY experiment) (Fig. 1B). In combination with the biogenic pathway, the orientation of H-5 and H-9 was assigned as *β*. In the ROESY spectrum (Fig. S6), the cross peaks observed between the proton pairs Me19/H-5 and H-9/H-12 indicated that H-5 and H-9 were on the same side towards *β*-orientation. The ROESY correlations of H-8/Me-20 suggested that H-8 and Me-20 took *α*-configuration. Thus, the relative configuration of compound **1** is determined as shown.

**Fig. 2:**
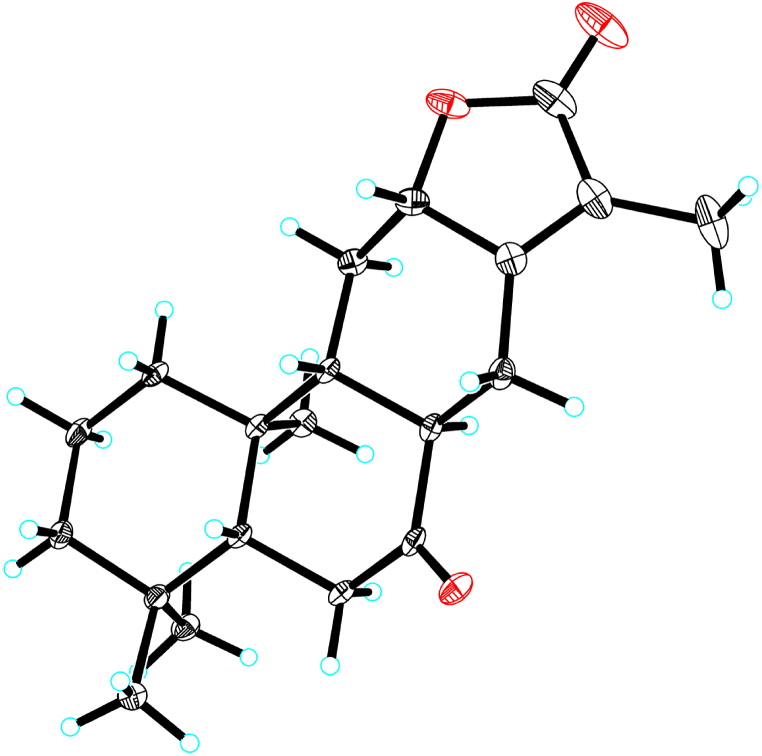
Single-crystal X-ray structure of **1**.

The absolute configuration of compound **1** was finally determined via single-crystal X-ray diffraction by using the anomalous dispersion of CuKα radiation; Flack parameter = 0.12(8); As shown in Fig. 2, the absolute configuration of compound **1** was unambiguously assigned as (12 S)-8*α*,14-dihydro-7-oxo-helioscopinolide A (Fig. 2, Fig. S7). Biogenetically, its 7-ketone moiety should be originated from precursor with 7-hydroxyl via oxidation [40].

### 3.2 Bioactive evaluation of compound **1**

#### 3.2.1 Compound **1** induces lysosome biogenesis

**Fig. 3:**
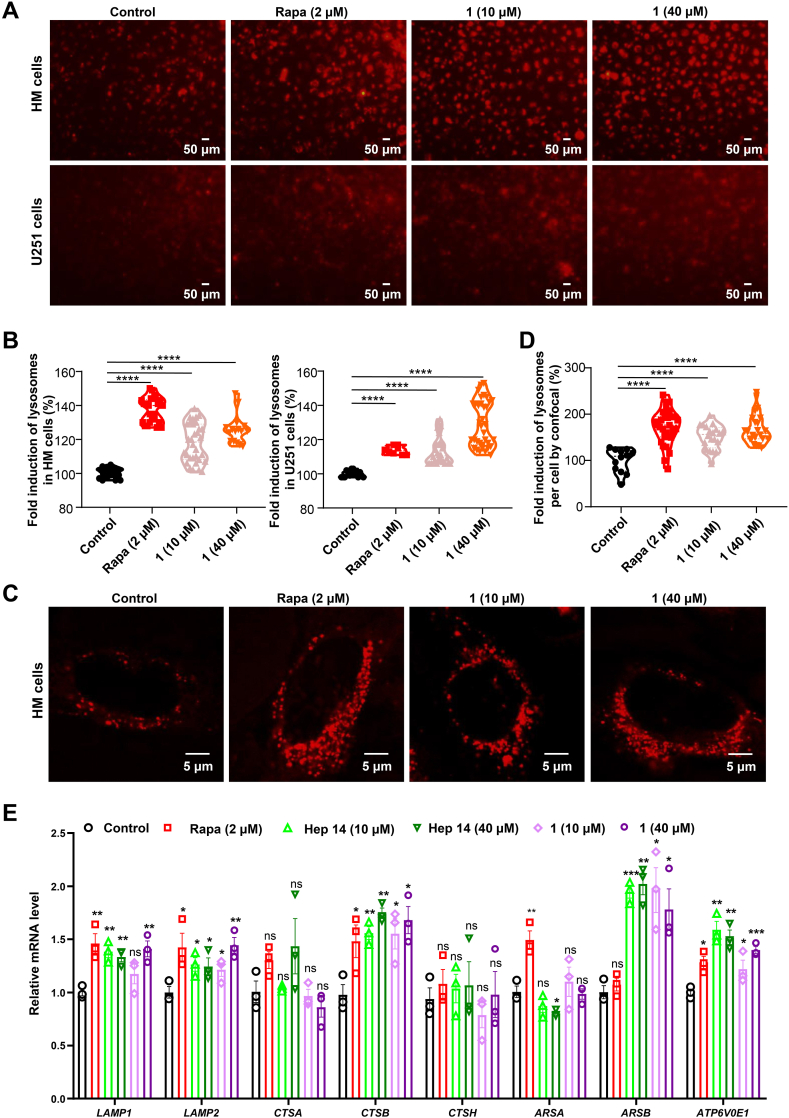
Compound **1** induces lysosome biogenesis A. Representative images of the HM and U251 cells with Lyso-Tracker Red staining after treatment with compound **1** (**1**, 10 μM and 40 μM) or rapamycin (Rapa, 2 μM). B. Quantifications of the fold change of lysosomes with different concentrations of **1** or rapa in A based on 3 independent experiments. C. Representative iamges of HM cells showing stained lysosomes by Lyso-Tracker Red after treatment with **1** (10 μM and 40 μM)**,** or Rapa (2 μM). D. Quantifications of the fold change of lysosome with different concentrations of **1** or rapa in C. E. The relative mRNA levels of lysosomal genes after the treatment of **1** (10 μM and 40 μM), Rapa (2 μM) or Hep 14 (10 μM and 40 μM) for 24 h *, *P* < 0.05; **, *P* < 0.01; ***, *P* < 0.001; ****, *P* < 0.0001; ns, not significant; one-way ANOVA with the Dunnett's *post hoc* test. Bars represent mean ± SD. (For interpretation of the references to color in this figure legend, the reader is referred to the Web version of this article.)

We treated human microglia (HM) and U251 cells with compound **1** and examined the change in lysosome number using Lyso-Tracker Red. Compound **1** induced a concentration-dependent increase in Lyso-Tracker Red staining and the intensities were 118% at 10 μM and 126% at 40 μM in HM cells, similar to that caused by the mTOR inhibitor Rapamycin at 137% intensity. The increasing effect was also observed in U251 cells with the intensities of 113% at 10 μM and 129% at 40 μM (Fig. 3A–B). In order to better observe the staining of lysosomes, HM cells were also cultured in the glass-bottom cell culture dish, and the individual cells were pictured under the confocal laser scanning microscope (Fig. 3C–D). Moreover, the mRNA levels of lysosomal genes, including lysosomal associated membrane protein 1 (*LAMP1*), *LAMP2*, ATPase H^+^ transporting V0 subunit E1 (*ATP6V0E1*), cathepsinB (*CTSB*), and arylsulfatase B (*ARSB*) were upregulated at 24 h after treatment with compound **1** in U251 cells, although the *CTSA* and *CTSH* were not significantly changed (Fig. 3E). We used the Hep 14 as an additional positive control [25]. These findings confirmed that compound **1** could induce lysosome biogenesis.

#### 3.2.2 Compound **1** has the activity of autophagy activation

**Fig. 4:**
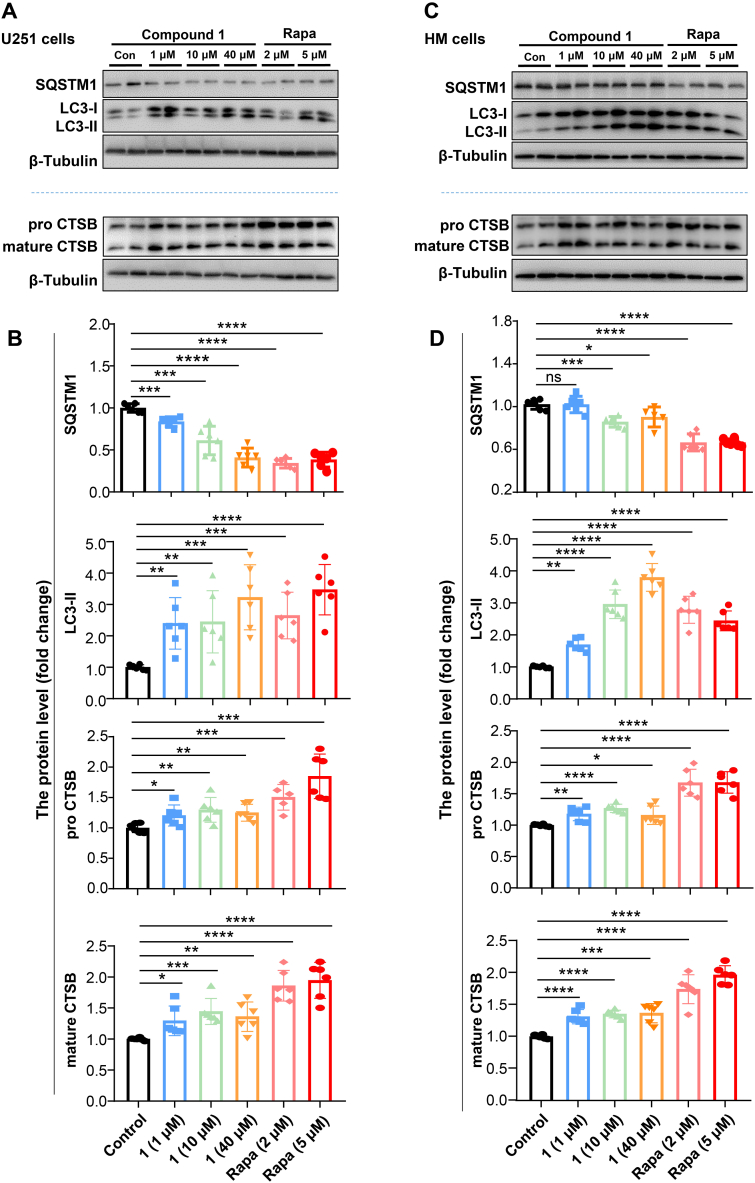
Compound **1** activates autophagy A. Western blot result shows the protein levels of CTSB (pro CTSB and mature CTSB), LC3 and SQSTM1 in the U251 cells treated with compound **1** (**1**, 1 μM, 10 μM and 40 μM) or rapamycin (Rapa, 2 μM and 5 μM). B. Quantifications of the protein levels of CTSB, SQSTM1 and LC3-II in the U251 cells in A based on 3 independent experiments. C. Western blot result shows the protein levels of CTSB, LC3 and SQSTM1 in the HM cells treated with **1** (1 μM, 10 μM and 40 μM) or Rapa (2 μM and 5 μM). D. Quantification of the protein levels of CTSB, SQSTM1 and LC3-II in the HM cells in C based on 3 independent experiments. *, *P* < 0.05; **, *P* < 0.01; ***, *P* < 0.001; ****, *P* < 0.0001; ns, not significant; one-way ANOVA with the Dunnett's *post hoc* test. Bars represent mean ± SD.

In order to test whether compound **1** would affect autophagy, we treated HM and U251 cells with compound **1**. An increased level of the lipidated (PE-conjugated) form of MAP1LC3/LC3 (microtubule-associated protein 1 light chain 3; LC3-II) and a decreased protein level of SQSTM1 (sequestosome 1) in a dose-dependent manner were observed in U251 cells treated with compound **1** (Fig. 4A–B). This observation could be confirmed in HM cells (Fig. 4C–D). Moreover, the protein level of CTSB (both pro CTSB and mature CTSB) was increased in response to compound **1**, consistent with the mRNA level in Fig. 3E.

**Fig. 5:**
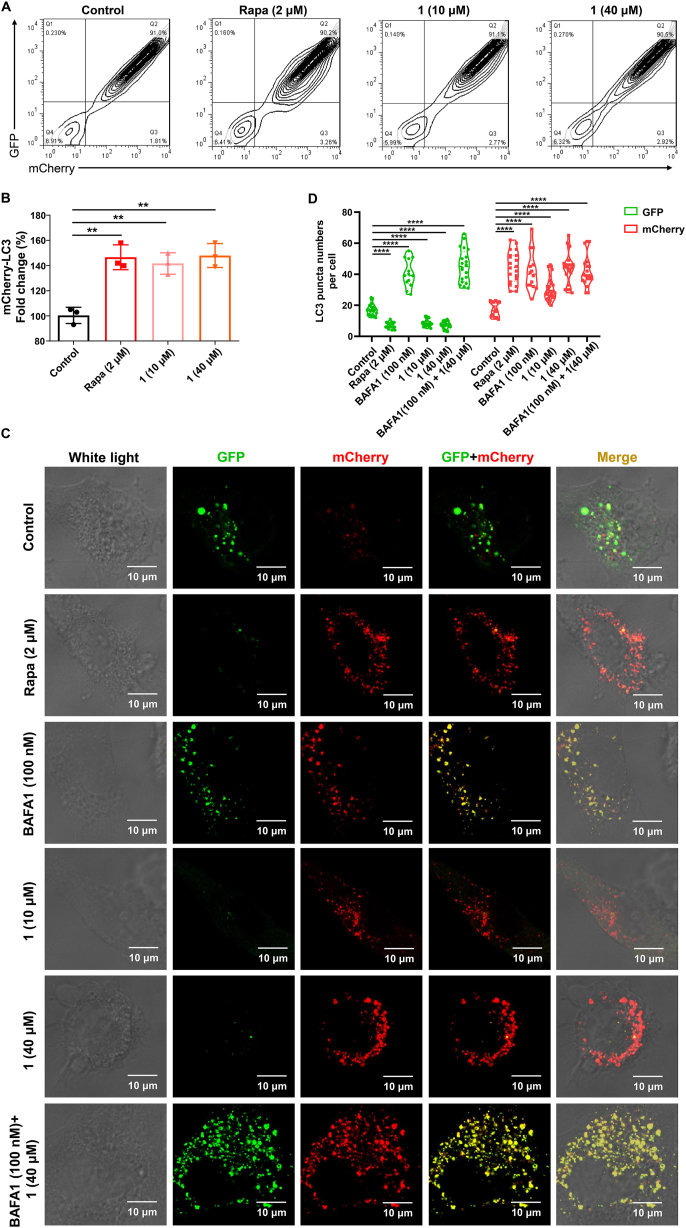
Compound **1** increases autophagic flux A. Flow cytometry of HM mCherry-GFP-LC3 cells with or without drug treatment. The percentage of 10,000 cells expressing GFP or/and mCherry were counted. The Q1 area represents the proportion of cells with green fluorescence; the Q2 area represents the proportion of cells with both red and green fluorescence; the Q3 area represents the proportion of cells with only red fluorescence; the Q4 area represents the proportion of cells showing no red and green fluorescene. B. Quantification of the Q3 area in A based on 3 independent experiments. Record the proportion of cells that only emit red fluorescence in Q3 area under each treatment. C. Representative images of HM mCherry-GFP-LC3 cells with treatment of compound **1** (**1**, 10 μM and 40 μM), rapamycin (Rapa, 2 μM) or/and Bafilomycin A1 (BAFA1, 100 nM). Scale bars, 10 μm. D. Quantification of the LC3 puncta in C. GFP for autophagosome and mCherry for autolysosome. Data shown are representative of 3 independent experiments. **, *P* < 0.01; ****, *P* < 0.0001; one-way ANOVA with the Dunnett's *post hoc* test. Bars represent mean ± SD. (For interpretation of the references to color in this figure legend, the reader is referred to the Web version of this article.)

We introduced the tandem monomeric mCherry-GFP-tagged LC3 (mCherry-GFP-LC3) reporter into HM to determine the effect of compound **1** on autophagic flux. The mCherry-GFP-LC3 in autolysosomes displayed more stable red mCherry fluorescence in the acidic lysosome while the GFP signal was sensitive to the acidic condition [41]. We used flow cytometry analysis, to verify whether the autophagic flux was enhanced in response to compound **1** treatment. We used DMSO (dimethyl sulfoxide, a solvent of compound **1**) as a negative control and rapamycin (an autophagy inducer) as a positive control. Treatment with compound **1** significantly increased the autophagic flux (intensity by 142% at 10 μM and 148% at 40 μM) (Fig. 5A–B), and this result was further confirmed by confocal analysis (Fig. 5C). We observed an increased number of red puncta and a decreased number of green puncta in the rapamycin-treated HM mCherry-GFP-LC3 cells (Fig. 5C–D), indicating increased autophagic flux. Treatment with compound **1** had a similar effect as rapamycin. We used BAFA1 (bafilomycin A1), an inhibitor of the vacuolar (V)-type ATPase that results in blockage of autophagosome-lysosome fusion [42], to further show the role of autophagy induced by compound **1**. We found that both of the green and red puncta numbers are increased after BAFA1 treatment (Fig. 5C–D), as the autophagosome-lysosome fusion process was blocked by BAFA1. Moreover, the effect of increased autophagic flux by compound **1** was abolished by BAFA1 (Fig. 5C–D). Collectively, these results demonstrated that compound **1** has the activity to activate ALP.

#### 3.2.3 Compound **1** induces the nuclear translocation of TFEB

**Fig. 6:**
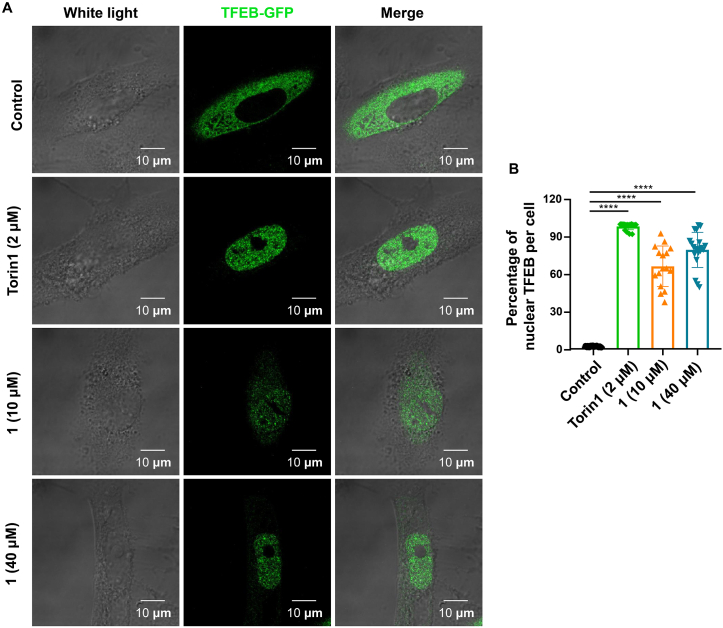
Compound **1** induces the nuclear translocation of TFEB. A. Representative images of subcellular locations of TFEB in HM TFEB-GFP cells treated with compound **1** (10 μM and 40 μM) or Torin1 (2 μM) for 6 h. Scale bars, 10 μm. B. Quantification of nuclear translocation of TFEB-GFP in A based on 3 independent experiments. ****, *P* < 0.0001; one-way ANOVA with the Dunnett's *post hoc* test. Bars represent mean ± SD.

Next, we further investigated the mechanism by which compound **1** regulates ALP. As transcription factor EB (TFEB) is a master regulator in regulating lysosomal biogenesis and autophagy [[43], [44], [45], [46]], we further tested whether compound **1** could induce the translocation of nuclear TFEB. We used HM TFEB-GFP cells (HM cells with the stably expressing TFEB-GFP). In this assay, we used the Torin1 as a positive control [25]. We found that the nuclear translocation of TFEB was significantly increased after compound **1** treatment for 6 h (Fig. 6A), with the intensity of 63% at 10 μM and 78% at 40 μM, compared to that of control at 2.6% (Fig. 6B).

### 3.3 Structure-activity relationship

**Fig. 7:**
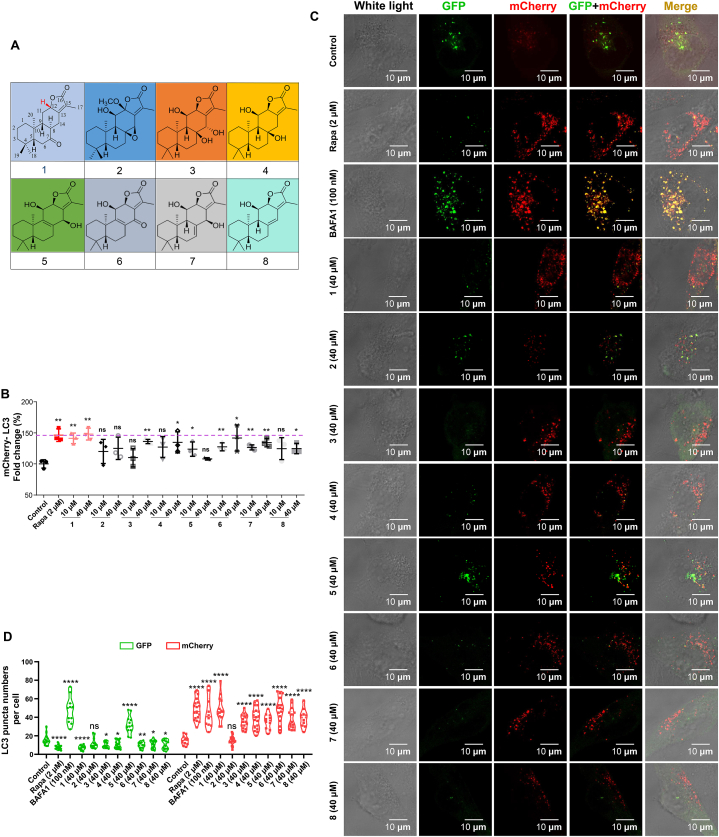
The structures of compound **1** and other seven abietane diterpenes and their activities in activating autophagic flux A. The structure of compound **1** and other seven abietane diterpenes (**2**–**8**). B. The fold change of autophagic flux in HM mCherry-GFP-LC3 cells with the treatment of compound **1** and other compounds (**2**–**8**), respectively. C. Representative images of HM mCherry-GFP-LC3 cells with treatment of **1**–**8** at the concentrations of 40 μM, respectively. Scale bars, 10 μm. D. Quantification of the LC3 puncta in C based on 3 independent experiments. Rapa and BAFA1 as the positive controls. ns, not significant; *, *P* < 0.05; **, *P* < 0.01; ****, *P* < 0.0001; one-way ANOVA with the Dunnett's *post hoc* test. Bars represent mean ± SD.

In order to analyze the structure-activity relationship, we performed flow cytometry analysis on other *ent*-abietane diterpenes (**2**–**8**) (Fig. 7A–B) from *Euphorbia ebracteolata* with HM mCherry-GFP-LC3 cells [47]. These compound **2**–**8** had a lower capacity (intensity by 125%, 136%, 134%, 108%, 141%, 134% and 124% at 40 μM, respectively) in activating autophagic flux than that of **1** (intensity by 148% at 40 μM). In addition, these results were further confirmed by confocal analysis (Fig. 7**C**-**D**). The SAR analyses suggested that the carbonyl at C-7 in **1** as a nucleophilic group might be a key active group, although we could not rule out a possibility that the orientation of *α*,*β*-unsaturated *δ*-lactone affects the occurrence of autophagy.

## 4 Conclusions

In this study, we isolated compound **1**, a new *ent*-abietane lactone, isolated from *E. peplus* and determined its structure by spectroscopic analyses and single-crystal X-ray diffraction analysis. The potential bioactivity of compound **1** in activating ALP was TFEB-mediated based on the evidence below: 1) **1** induces lysosome biogenesis by Lyso-Tracker Red staining and qRT-PCR; 2) **1** increases ALP at the protein levels such as increasing CTSB, LC3-II and decreasing SQSTM1; 3) **1** increases autophagic flux in HM mCherry-GFP-LC3 cells by flow cytometry analysis and confocal analysis which provides more intuitive evidence with decreasing green puncta and increasing red puncta, and this effect was abolished by BAFA1; 4) **1** activates the translocate of TFEB from the cytoplasm to the nucleus. The SAR analyses implied that the carbonyl at C-7 in **1** might be a key active group. Disruption of the ALP is a pathological hallmark in many human diseases [1,[48], [49], [50], [51], [52], [53], [54]]. Overall, this study is relatively preliminary, further well-designed *in vitro* and *in vivo* studies are needed to determine whether compound **1** could be used for treating ALP-related neurodegenerative diseases in the future.

## Declarations

### Author contribution statement

Xiaoqian Ran: Performed the experiments; Analyzed and interpreted the data; Wrote the paper. Qing-yun Lu, Ying-Yao Li, Xue-Xue Pu, Yarong Guo, Ming-Rui Yuan, Shi-Peng Guan, Mao Sun: Performed the experiments; Analyzed and interpreted the data. Lijin Jiao: Analyzed and interpreted the data; Wrote the paper. Yong-Gang Yao, Xiao-Jiang Hao: Contributed reagents, materials, analysis tools or data; Wrote the paper. Ying-Tong Di: Conceived and designed the experiments; Wrote the paper. Rongcan Luo: Conceived and designed the experiments; Wrote the paper.

### Funding statement

Dr. Rongcan Luo was supported by the National Natural Science Foundation of China [31270988 and 31900737]; the Science and Technology Program of Yunnan Province [202201AW070010 and 202001AT070107]; the Youth Innovation Promotion Association of CAS [2021000011]; the Young Scientific and Technological Talents Promotion Project of Yunnan Association for science and Technology [2022000043]; the Open Project from Key Laboratory of Animal Models and Human Disease Mechanisms of the Chinese Academy of Sciences & Yunnan Province [AMHD-2021-4 and AMHD-2022-4].

Dr. Ying-Tong Di was supported by the 10.13039/501100001809National Natural Science Foundation of China [31770392].

Dr. Yong-Gang Yao was supported by the Science and Technology Program of Yunnan Province [202003AD150009]; the 10.13039/501100001809National Natural Science Foundation of China [31730037]; the Strategic Priority Research Program (B) of 10.13039/501100002367CAS [XDB02020003]; the Project for International Collaboration of the Bureau of International Collaboration, 10.13039/501100002367CAS [GJHZ1846].

### Data availability statement

Data will be made available on request.

### Declaration of interest's statement

The authors declare no conflict of interest.
