# Supplementary material for: Euphejolkinolide A, a new ent-abietane lactone from Euphorbia peplus L. with promising biological activity in activating the autophagy-lysosomal pathway
Source: Heliyon. 2023 Feb 13;9(2):e13691. doi: 10.1016/j.heliyon.2023.e13691 (PMC9958456; doi:10.1016/j.heliyon.2023.e13691)
Supplement: Multimedia component 1 [file mmc1.docx]

**Supporting Information**

**Euphejolkinolide A, a new *ent*-abietane lactone from *Euphorbia peplus* L. with promising biological activity in activating the autophagy-lysosomal pathway**

Xiaoqian Ran ^a,c,‡^, Qing-yun Lu ^b,‡^, Ying-Yao Li ^d^, Xue-Xue Pu ^e^, Yarong Guo ^a,f^, Ming-Rui Yuan ^b^, Shi-Peng Guan ^e^, Mao Sun ^b^, Lijin Jiao ^a,c^, Yong-Gang Yao ^a,c,g^, Ying-Tong Di ^b,c,e,*^, Xiao-Jiang Hao ^b,h,i^, and Rongcan Luo ^a,c,*^

^a^ Key Laboratory of Animal Models and Human Disease Mechanisms of the Chinese Academy of Sciences & Yunnan Province, KIZ-CUHK Joint Laboratory of Bioresources and Molecular Research in Common Diseases, Kunming Institute of Zoology, Chinese Academy of Sciences, Kunming 650204, China.

^b^ State Key Laboratory of Phytochemistry and Plant Resources in West China, Kunming Institute of Botany, Chinese Academy of Sciences, Kunming 650201, China.

^c^ Kunming College of Life Science, University of Chinese Academy of Sciences, Kunming, Yunnan 650204, China

^d^ College of Life Sciences, Yunnan University, Kunming 650091, China

^e^ College of Traditional Medicine, Yunnan University of Chinese Medicine, Kunming, 650500, China

^f^ School of Life Sciences, Division of Life Sciences and Medicine, University of Science and Technology of China, Hefei 230026, China

^g^ CAS Center for Excellence in Brain Science and Intelligence Technology, Chinese Academy of Sciences, Shanghai 200031, China

^h^ Yunnan Key Laboratory of Natural Medicinal Chemistry, Kunming, Yunnan 650201, China

^i^ Guizhou Chemical Drug Research and Development Engineering Technical Center, Guizhou Medicinal University, Guiyang 550004, China

‡ These authors contributed equally to this work.

* Corresponding authors

* Email: diyt@mail.kib.ac.cn.

* Email: luorongcan@mail.kiz.ac.cn.

**List of Contents**

[**Figure S1** ^1^H-NMR spectrum of compound **1**. 4](#_Toc126413230)

[**Figure S2** ^13^C-NMR spectrum of compound **1**. 7](#_Toc126413231)

[**Figure S3 ^1^H-^1^H** COSY spectrum of compound **1**. 10](#_Toc126413232)

[**Figure S4** HSQC spectrum of compound **1**. 14](#_Toc126413233)

[**Figure S5** HMBC spectrum of compound **1**. 17](#_Toc126413234)

[**Figure S6** ROESY spectrum of compound **1**. 20](#_Toc126413235)

[**Figure S7** Single-crystal X-ray structure of compound **1**. 21](#_Toc126413236)

[**Figure S8** CD spectrum of compound **1**. 22](#_Toc126413237)

[**Figure S9** UV spectrum of compound **1**. 23](#_Toc126413238)

[**Figure S10** IR spectrum of compound **1**. 24](#_Toc126413239)

[**Figure S11** HR-ESI-MS spectrum of compound **1**. 25](#_Toc126413240)

[**Figure S12** Uncropped images of western blot. 26](#_Toc126413241)

[**Table S1** Crystal data and structure refinement for compound **1**. 27](#_Toc126413242)

[**Table S2** qRT-PCR primer pairs for measuring mRNA levels of the targeted genes in this atudy 28](#_Toc126413243)

Current Data Parameters

NAME **Euphejolkinolide A (1)**

EXPNO 21

PROCNO 1

F2 - Acquisition Parameters

INSTRUM spect PROBHD 5 mm CPQCI 1H/

PULPROG zg TD 65536

SOLVENT CDCl_3_ NS 1

DS 0 SWH 10000.000 Hz

FIDRES 0.152588 Hz AQ 3.2767999 sec

RG 21.83 DW 50.000 usec

DE 10.00 usec TE 298.2 K

D1 1.00000000 sec TD0 1

======== CHANNEL f1 ========

SFO1 500.1830011 MHz NUC1 1H

P1 10.80 usec PLW1 4.37519979 W

F2 - Processing parameters

SI 65536 SF 500.1800150 MHz

WDW EM SSB 0

LB 0.10 Hz GB 0

PC 1.00

**Figure S1** ^1^H-NMR spectrum of compound **1**.

Current Data Parameters

NAME **Euphejolkinolide A (1)**

EXPNO 22

PROCNO 1

F2 - Acquisition Parameters

INSTRUM spect PROBHD 5 mm CPQCI 1H/

PULPROG zgdc TD 65536

SOLVENT CDCl_3_ NS 32

DS 4 SWH 29761.904 Hz

FIDRES 0.454131 Hz AQ 1.1010048 sec

RG 187.72 DW 16.800 usec

DE 18.00 usec TE 298.1 K

D1 3.00000000 sec D11 0.03000000 sec

TD0 1

======== CHANNEL f1 ========

SFO1 125.7844473 MHz NUC1 13C

P1 15.70 usec PLW1 125.00000000 W

======== CHANNEL f2 ========

SFO2 500.1827508 MHz NUC2 1H

CPDPRG[2 waltz16 PCPD2 100.00 usec

PLW2 4.37519979 W PLW12 0.05686000 W

F2 - Processing parameters

SI 32768 SF 125.7703754 MHz

WDW EM SSB 0

LB 1.00 Hz GB 0

PC 0.20

**Figure S2** ^13^C-NMR spectrum of compound **1**.

Current Data Parameters

NAME **Euphejolkinolide A (1)**

EXPNO 25

PROCNO 1

F2 - Acquisition Parameters

INSTRUM spect PROBHD 5 mm CPQCI 1H/

PULPROG cosygpmfqf TD 2048

SOLVENT CDCl_3_ NS 1

DS 8 SWH 5000.000 Hz

FIDRES 2.441406 Hz AQ 0.2048000 sec

RG 187.72 DW 100.000 usec

DE 10.00 usec TE 298.1 K

D0 0 sec D1 1.00000000 sec

D13 0.00000400 sec D16 0.00020000 sec

IN0 0.00020000 sec

======== CHANNEL f1 ========

SFO1 500.1820007 MHz NUC1 1H

P1 10.80 usec PLW1 4.37519979 W

====== GRADIENT CHANNEL =====

GPNAM[1] SMSQ10.100 GPNAM[2] SMSQ10.100

GPNAM[3] SMSQ10.100 GPZ1 16.00 %

GPZ2 12.00 % GPZ3 40.00 %

P16 1000.00 usec

F1 - Acquisition parameters

TD 128 SFO1 500.182 MHz

FIDRES 39.062500 Hz SW 9.996 ppm

FnMODE QF

F2 - Processing parameters

SI 1024 SF 500.1800130 MHz

WDW QSINE SSB 0

LB 0 Hz GB 0

PC 1.40

F1 - Processing parameters

SI 1024 MC2 QF

SF 500.1800130 MHz WDW QSINE

SSB 0 LB 0 Hz

GB 0

**Figure S3 ^1^H-^1^H** COSY spectrum of compound **1**.

Current Data Parameters

NAME **Euphejolkinolide A (1)**

EXPNO 26

PROCNO 1

F2 - Acquisition Parameters

INSTRUM spect PROBHD 5 mm CPQCI 1H/

PULPROG hsqcetgpsi2 TD 1024

SOLVENT CDCl_3_  NS 2

DS 16 SWH 5000.000 Hz

FIDRES 4.882813 Hz AQ 0.1024000 sec

RG 187.72 DW 100.000 usec

DE 10.00 usec TE 298.2 K

CNST2 145.0000000 D0 0.00000300 sec

D1 1.20000005 sec D4 0.00172414 sec

D11 0.03000000 sec D16 0.00020000 sec

D24 0.00086207 sec IN0 0.00002370 sec

ZGOPTNS

======== CHANNEL f1 ========

SFO1 500.1820007 MHz NUC1 1H

P1 10.80 usec P2 21.60 usec

P28 0 usec PLW1 4.37519979 W

======== CHANNEL f2 ========

SFO2 125.7806742 MHz NUC2 13C

CPDPRG[2 bi_p5m4sp_4sp.2 P3 11.90 usec

P4 23.80 usec P63 1500.00 usec

PLW2 175.00000000 W PLW12 5.86549997 W

SPNAM[14 Crp32,1.5,20.2 SPOAL14 0.500

SPOFFS14 0 Hz SPW14 16.15500069 W

SPNAM[31 Crp32,1.5,20.2 SPOAL31 0.500

SPOFFS31 0 Hz SPW31 4.03879976 W

====== GRADIENT CHANNEL =====

GPNAM[1] SMSQ10.100 GPNAM[2] SMSQ10.100

GPNAM[3] SMSQ10.100 GPNAM[4] SMSQ10.100

GPZ1 80.00 % GPZ2 20.10 %

GPZ3 11.00 % GPZ4 -5.00 %

P16 1000.00 usec P19 600.00 usec

F1 - Acquisition parameters

TD 148 SFO1 125.7807 MHz

FIDRES 142.547607 Hz SW 167.729 ppm

FnMODE Echo-Antiecho

F2 - Processing parameters

SI 1024 SF 500.1800130 MHz

WDW QSINE SSB 2

LB 0 Hz GB 0

PC 1.40

F1 - Processing parameters

SI 1024 MC2 echo-antiecho

SF 125.7703610 MHz WDW QSINE

SSB 2 LB 0 Hz

GB 0

**Figure S4** HSQC spectrum of compound **1**.

Current Data Parameters

NAME **Euphejolkinolide A (1)**

EXPNO 27

PROCNO 1

F2 - Acquisition Parameters

INSTRUM spect PROBHD 5 mm CPQCI 1H/

PULPROG hmbcgplpndqf TD 2048

SOLVENT CDCl_3_ NS 8

DS 16 SWH 5000.000 Hz

FIDRES 2.441406 Hz AQ 0.2048000 sec

RG 187.72 DW 100.000 usec

DE 10.00 usec TE 298.2 K

CNST2 145.0000000 CNST13 6.0000000

D0 0.00000300 sec D1 1.85699201 sec

D2 0.00344828 sec D6 0.08333334 sec

D16 0.00020000 sec IN0 0.00001740 sec

======== CHANNEL f1 ========

SFO1 500.1820007 MHz NUC1 1H

P1 10.80 usec P2 21.60 usec

PLW1 4.37519979 W

======== CHANNEL f2 ========

SFO2 125.7845731 MHz NUC2 13C

P3 11.90 usec PLW2 175.00000000 W

====== GRADIENT CHANNEL =====

GPNAM[1] SMSQ10.100 GPNAM[2] SMSQ10.100

GPNAM[3] SMSQ10.100 GPZ1 50.00 %

GPZ2 30.00 % GPZ3 40.10 %

P16 1000.00 usec

F1 - Acquisition parameters

TD 128 SFO1 125.7846 MHz

FIDRES 224.497131 Hz SW 228.451 ppm

FnMODE QF

F2 - Processing parameters

SI 2048 SF 500.1800130 MHz

WDW SINE SSB 0

LB 0 Hz GB 0

PC 1.40

F1 - Processing parameters

SI 1024 MC2 QF

SF 125.7703610 MHz WDW SINE

SSB 0 LB 0 Hz

GB 0

**Figure S5** HMBC spectrum of compound **1**.

Current Data Parameters

NAME **Euphejolkinolide A (1)**

EXPNO 28

PROCNO 1

F2 - Acquisition Parameters

INSTRUM spect PROBHD 5 mm CPQCI 1H/

PULPROG croesyph TD 1024

SOLVENT CDCl_3_ NS 8

DS 32 SWH 5000.000 Hz

FIDRES 4.882813 Hz AQ 0.1024000 sec

RG 2.01 DW 100.000 usec

DE 10.00 usec TE 298.1 K

D0 0.00008625 sec D1 1.20000005 sec

D12 0.00002000 sec D13 0.00000400 sec

IN0 0.00020000 sec

======== CHANNEL f1 ========

SFO1 500.1820007 MHz NUC1 1H

P1 10.80 usec P15 1200000.00 usec

PLW1 4.37519979 W PLW11 0.06300300 W

F1 - Acquisition parameters

TD 160 SFO1 500.182 MHz

FIDRES 31.250000 Hz SW 9.996 ppm

FnMODE States-TPPI

F2 - Processing parameters

SI 1024 SF 500.1800130 MHz

WDW QSINE SSB 2

LB 0 Hz GB 0

PC 1.00

F1 - Processing parameters

SI 1024 MC2 States-TPPI

SF 500.1800130 MHz WDW QSINE

SSB 2 LB 0 Hz

GB 0

**Figure S6** ROESY spectrum of compound **1**.

**
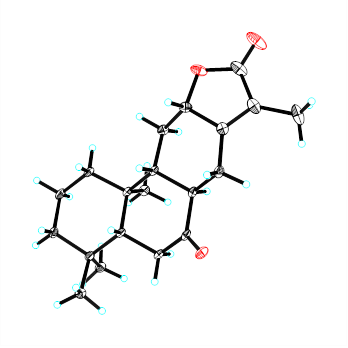

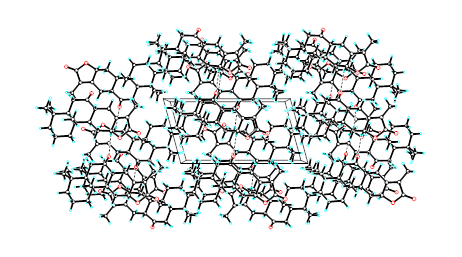
**

**Figure S7** Single-crystal X-ray structure of compound **1**.


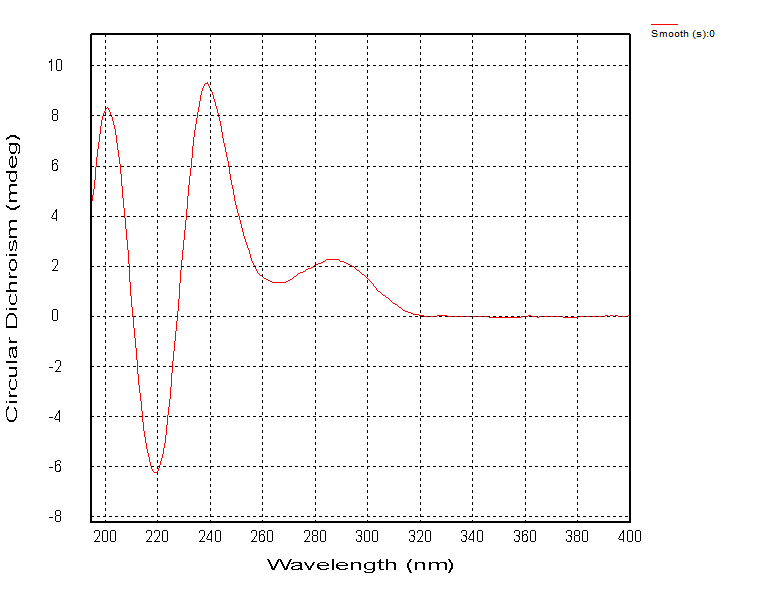


**Figure S8** CD spectrum of compound **1**.


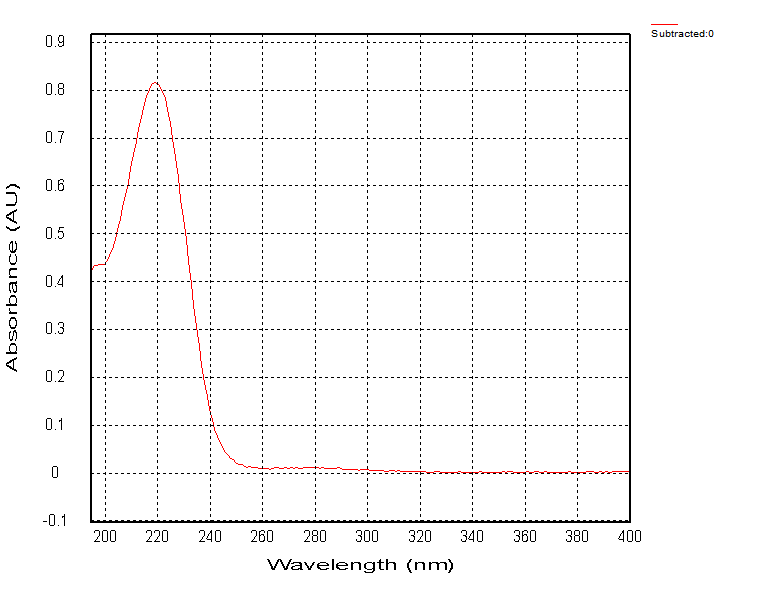


**Figure S9** UV spectrum of compound **1**.

**Figure S10** IR spectrum of compound **1**.

**
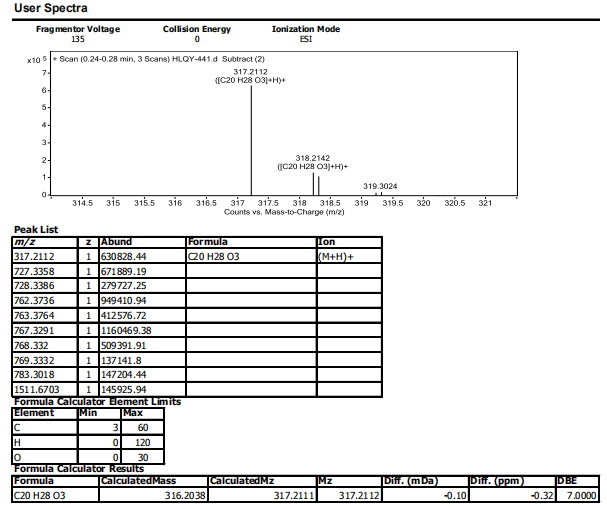
**

**Figure S11** HR-ESI-MS spectrum of compound **1**.


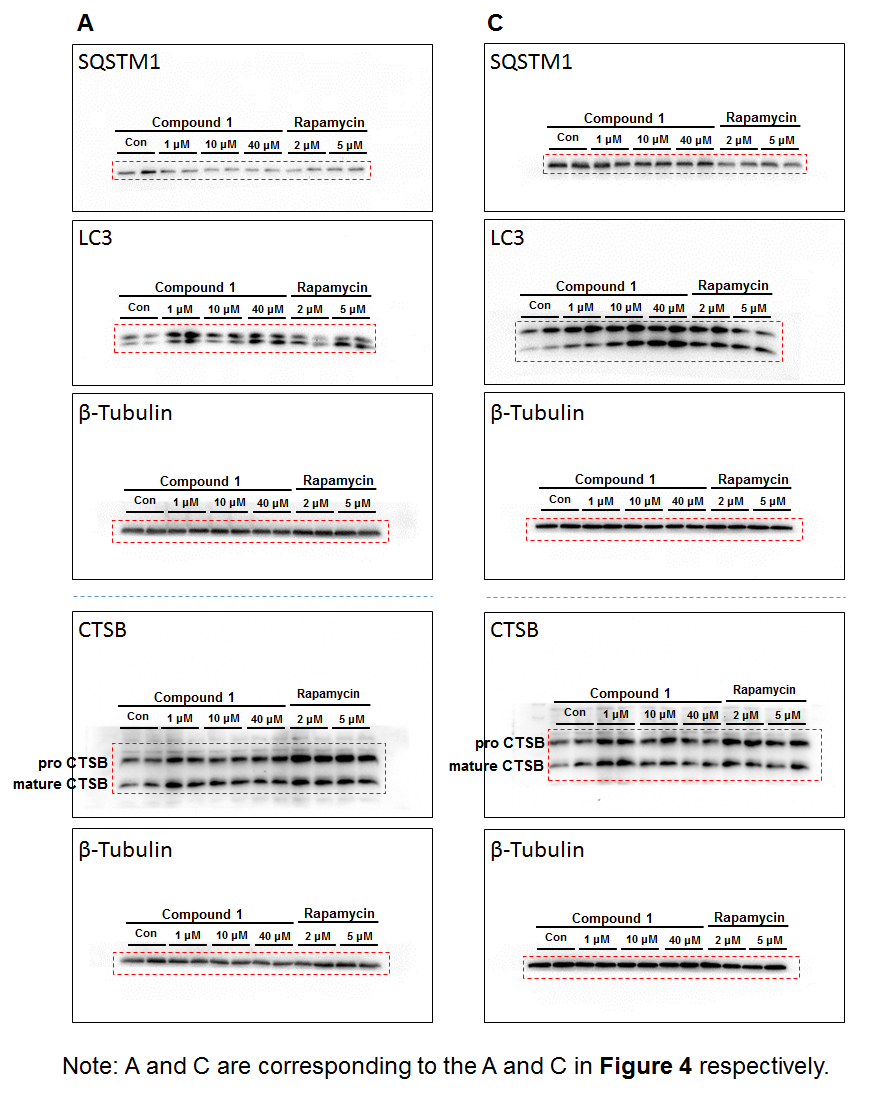


**Figure S12** Uncropped images of western blot.

**Table S1** Crystal data and structure refinement for compound **1**.

| Empirical formula | C_20_ H_28_ O_3_ |
| --- | --- |
| Formula weight | 316.42 |
| Temperature | 100(2) K |
| Wavelength | 1.54178 Å |
| Crystal system | Monoclinic |
| Space group | P2**_1_**2**_1_**2**_1_** |
| Unit cell dimensions | a = 7.1902(2) Å α= 90°. |
|  | b = 9.1117(2) Å β= 105.2350(10)°. |
|  | c = 13.5961(3) Å γ = 90°. |
| Volume | 859.44(4) Å3 |
| Z | 2 |
| Density (calculated) | 1.223 Mg/m3 |
| Absorption coefficient | 0.635 mm-1 |
| F(000) | 344 |
| Crystal size | 0.400 x 0.320 x 0.090 mm3 |
| Theta range for data collection | 5.91 to 72.16°. |
| Index ranges | -8<=h<=8, -11<=k<=11, -16<=l<=16 |
| Reflections collected | 15218 |
| Independent reflections | 3381 [R(int) = 0.0424] |
| Completeness to theta = 72.16° | 99.8 % |
| Absorption correction | Semi-empirical from equivalents |
| Max. and min. transmission | 0.94 and 0.76 |
| Refinement method | Full-matrix least-squares on F2 |
| Data / restraints / parameters | 3381 / 1 / 212 |
| Final R indices [I>2sigma(I)] | R1 = 0.0304, wR2 = 0.0802 |
| R indices (all data) | R1 = 0.0312, wR2 = 0.0813 |
| Absolute structure parameter | 0.12(8) |
| Largest diff. peak and hole | 0.195 and -0.205 e.Å-3 |

**Table S2** qRT-PCR primer pairs for measuring mRNA levels of the targeted genes in this atudy

| **Primer** | **Sequence (5'-3')** | **Product length (bp)** |
| --- | --- | --- |
| *CTSB Forward* | ACAACGTGGACATGAGCTACT | 85 |
| *CTSB Reverse* | TCGGTAAACATAACTCTCTGGGG |  |
| *CTSA Forward* | TCCCAGCATGAACCTTCAGG | 89 |
| *CTSA Reverse* | AGTAGGCAAAGTAGACCAGGG |  |
| *CTSH Forward* | AAACGCCCACAACAATGGGA | 122 |
| *CTSH Reverse* | TGGTGGCTGAGCAATTCTGAG |  |
| *ARSB Forward* | TCTTGCTGGCAGACGACCTA | 121 |
| *ARSB Reverse* | GGCTGCGTGTAGTAGTTGTCC |  |
| *ARSA Forward* | CACACCCACTACCCTCAGTTC | 126 |
| *ARSA Reverse* | CAGGTCCCCTATGGCTGTC |  |
| *ATP6V0E1 Forward* | GTCCTAACCGGGGAGTTATCA | 101 |
| *ATP6V0E1 Reverse* | AAAGAGAGGGTTGAGTTGGGC |  |
| *LAMP1 Forward* | TCTCAGTGAACTACGACACCA | 151 |
| *LAMP1 Reverse* | AGTGTATGTCCTCTTCCAAAAGC |  |
| *LAMP2 Forward* | GAAAATGCCACTTGCCTTTATGC | 184 |
| *LAMP2 Reverse* | AGGAAAAGCCAGGTCCGAAC |  |
| *GAPDH Forward* | GGAGCGAGATCCCTCCAAAAT | 101 |
| *GAPDH Reverse* | GGCTGTTGTCATACTTCTCATGG |  |
